# Prognostic Stratification Based on a Novel Nomogram for Solitary Large Hepatocellular Carcinoma After Curative Resection

**DOI:** 10.3389/fonc.2020.556489

**Published:** 2020-11-16

**Authors:** Hongkai Zhuang, Zixuan Zhou, Zuyi Ma, Shanzhou Huang, Yuanfeng Gong, Zedan Zhang, Baohua Hou, Weixuan Yu, Chuanzhao Zhang

**Affiliations:** ^1^Department of General Surgery, Guangdong Provincial People's Hospital, Guangdong Academy of Medical Sciences, Guangzhou, China; ^2^Shantou University of Medical College, Shantou, China; ^3^The Second School of Clinical Medicine, Southern Medical University, Guangzhou, China; ^4^Department of General Surgery, Tungwah Hospital of Sun Yat-Sen University, Dongguan, China

**Keywords:** solitary larger hepatocellular carcinoma, nomogram, overall survival, recurrence-free survival, transarterial chemoembolization, radiofrequency ablation

## Abstract

Solitary large hepatocellular carcinoma (SLHCC) is a specific subtype of HCC with unique characteristics. It is of great interest to assess and stratify the prognosis of SLHCCs after curative resection. In this study, we tried to construct a prognostic nomogram for SLHCC following curative resection through a retrospective analysis of 202 SLHCC cases. Seven prognostic factors were identified and integrated to establish a novel prognostic nomogram, which included tumor size, microvascular invasion, tumor differentiation, Ki67 (%), α-fetoprotein (AFP), carbohydrate antigen 125 (CA125), and HBsAg status. The Harrell's concordance index (C-index) of the nomogram for overall survival (OS) in the training, validation, and whole sets was 0.752, 0.703, and 0.733, respectively. Furthermore, the area under the curve (AUC) of the receiver operating characteristic (ROC) curve of the nomogram for predicting 1-, 3-, and 5-year OS indicated that the nomogram had an optimal discrimination of the prognostic prediction for SLHCC. The total score of each patient was calculated based on the nomogram, and patients were divided into three subgroups: low-risk group (total score ≦ 107), medium-risk group (107 < total score ≤ 125), and high-risk group (total score > 125). The 1-, 3-, and 5-year OS rates of the low-risk, medium-risk, and high-risk groups in the whole set were 89.3 vs. 70.1 vs. 33.3%, 76.6 vs. 37.8 vs. 14.5%, and 69.8 vs. 25.1 vs. 12.5%, respectively (*P* < 0.001). Similar results were shown in terms of the recurrence-free survival (RFS) rate. By analyzing 101 cases of recurrent tumors, transarterial chemoembolization (TACE) plus radiofrequency ablation (RFA)/surgery was found to prolong patient survival when compared to TACE alone in the low-risk group, but not in the medium/high-risk group. In conclusion, our prognostic nomogram successfully stratifies the prognosis for SLHCC after curative resection, which deserves further study in future clinical practice.

## Introduction

As the most common primary liver malignancy worldwide, hepatocellular carcinoma (HCC) ranks as the second leading cause of cancer-related death and has increasing incidence (1). The Barcelona Clinic Liver Cancer (BCLC) system is a very practical staging system for HCC, which mainly focuses on tumor size and number, Child–Pugh score, and the Eastern Cooperative Oncology Group (ECOG) performance status, to determine tumor stage and treatment strategies (2). All solitary tumors ≥2 cm were designated as stage A in the updated BCLC staging system (3). For stage A patients with preserved liver function and good performance status, surgical resection or liver transplantation is the recommended curative treatment (2–5).

Among stage A tumors, solitary large HCC (SLHCC) with a diameter >5 cm is worthy of note because of its unique characteristics (6). For example, large HCCs are associated with a higher risk of microvascular invasion (MVI), which is a critical oncological predictor for poor prognosis (7–9). Tsilimigras et al. subclassified SLHCC as BCLC A1 and found that the prognosis of patients with SLHCC after curative resection was similar to that among patients presenting with BCLC-B HCCs (two or three tumors ≥3 cm or ≥ four tumors) (10). Indeed, different outcomes were reported for SLHCC patients following curative resection, which may be due to tumor heterogeneity (11–13). Therefore, it is of great interest to develop an accurate prognostic nomogram to stratify the prognosis of SLHCC patients after curative resection.

To our knowledge, a prognostic nomogram for SLHCC following curative resection has not yet been studied. In the current study, we retrospectively collected the data of SLHCC patients after curative resection in our institution and performed univariate Cox regression analysis and least absolute shrinkage and selection operator (LASSO) regression analysis to identify prognostic factors for SLHCC. Further, we developed and validated a novel nomogram based on these prognostic factors.

## Materials and Methods

### Patient Data

We retrospectively collected and analyzed the data of 202 patients who had an SLHCC (tumor diameter > 5 cm) after R0 resection in Guangdong Provincial People's Hospital between 2008 and 2016. All patients received optimal postoperative therapy provided by multiple disciplinary teams by synthesizing the patients' tumor burden and physical condition. The exclusion criteria included: (a) loss of follow-up within 1 year after surgery; (b) patients with macrovascular invasion or distant metastatic disease; (c) patients who received preoperative anti-inflammatory treatments; (d) patients with preoperative infection, hematological, or inflammatory diseases; and (e) patients with a history of other malignancies. The protocol for this study was approved by the Clinical Research Ethics Committee of Guangdong Provincial People's Hospital, and informed consent was obtained.

The patients' clinicopathological parameters were extracted from medical records, including age, gender, histology of cirrhosis, tumor size, hepatic capsular invasion, microvascular invasion, tumor differentiation (Grades I/II/III/IV), Ki67 (%), neutrophil, lymphocyte, platelet, neutrophil-to-lymphocyte ratio (NLR), platelet-to-lymphocyte ratio (PLR), carbohydrate antigen 125 (CA125), carbohydrate antigen 199 (CA199), and HBsAg status. The Ki67 index (%) was determined by dividing the immunohistochemically stained tumor cells by nuclear-stained tumor cells. For further analysis, the enrolled patients were randomly grouped into a training set (*n* = 122) and a validation set (*n* = 80).

### Follow-Up

All patients with HCC were followed-up at our institution every 3 months for the first 2 years, every 6 months for the next 3 years, and once a year thereafter. Patients routinely received enhanced abdominal computed tomography (CT) scans or magnetic resonance imaging (MRI) every 3 months within the first year and every 6–12 months thereafter. OS was defined as the period from the date of operation to the date of death or final follow-up. RFS was defined as the period from the date of operation to the date of tumor relapse or final follow-up.

### Statistical Analysis

SPSS 25.0 software and R 3.5.2 project (http://www.r-project.org/) were used for analysis. The X-tile 3.6.1 software (Yale University, New Haven, CT, USA) (14) was used to calculate the optimal cutoff for continuous clinicopathological parameters, including age, tumor size, Ki67 (%), neutrophil, lymphocyte, platelet, NLR, PLR, α-fetoprotein (AFP), CA125, and CA199. The chi-squared test or Fisher's exact test was used to compare the clinicopathological parameters among the training, validation, and whole sets.

Univariate Cox regression analysis was used to determine prognostic parameters for the OS of SLHCC patients in the training set. Hazard ratio and 95% confidence interval (95% CI) were calculated. Then, among the parameters that were significant in the univariate Cox regression analysis, the key prognostic parameters for OS of SLHCC were further selected by LASSO regression analysis using the R package glmnet. Finally, a nomogram consisting of the key prognostic parameter selected from LASSO regression analysis was constructed using multivariate Cox regression analysis.

The predictive performance of the nomogram in the training set was assessed using the C-index, calibration curve, and AUC of the ROC curve (15). To further evaluate the predictive efficiency of the nomogram, the total scores of each patient in the validation and whole sets were calculated. The scores were used as a single factor to complete Cox regression of the validation and whole sets. Finally, the C-index, calibration curves, and ROC curves were obtained based on the Cox regression analysis.

Furthermore, we divided the patients in the training set into three subgroups (low-risk, medium-risk, and high-risk) according to the optimal cutoff points obtained from the X-tile 3.6.1 software. Patients in the validation and whole sets were also divided into low-, medium-, and high-risk groups according to the same optimal cutoff points in the training set. The 1-, 3-, and 5-year OS rates of each group were calculated, and Kaplan–Meier (KM) survival curves were performed. For all analyses, a value of *P* < 0.05 was considered statistically significant.

## Results

### Patient Clinicopathological Characteristics

A total of 202 patients with SLHCC were enrolled in the study. The detailed clinicopathological parameters and optimal cutoff values are summarized in Table 1. The median age of the patients was 53 (range 15–82) years, and 88.6% of the patients were male. The median follow-up time period was 56 months (95% CI, 52.1–59.9) and ranged from 1 to 120 months. The median OS and RFS were 36.0 (95% CI, 25.0–47.0) months and 31.0 (95% CI, 14.3–47.7) months, respectively. The 1-, 3-, and 5-year OS rates were 68.8, 50.0, and 41.0%, while the 1-, 2-, and 3-year RFS rates were 60.5, 52.6, and 47.7%, respectively. During the follow-up period, 121 (59.9%) patients died, and 101 (50.0%) patients had a recurrence before the last follow-up. Meanwhile, we found that all clinicopathological parameters were not significantly different between the training and validation sets (Table 1).

**Table 1:** Comparison of clinicopathological parameters between the training and validation sets.

	**Whole set (*n* = 202)**	**Training set (*n* = 122)**	**Validation set (*n* = 80)**	**Chi value**	***P*-value**
Age (≦50/>50 years)	150/52	94/28	56/24	1.256	0.534
Gender (female/male)	23/179	17/105	6/74	1.983	0.371
Cirrhosis (no/yes)	137/65	82/40	55/25	0.052	0.974
Preoperative TACE (no/yes)	190/12	114/8	76/4	0.210	0.90
Tumor size (≦7.1/>7.1 cm)	78/124	50/72	28/52	0.73	0.694
Hepatic capsular invasion (no/invasion but not breaking through/breaking through)	122/73/7	80/36/6	42/37/1	6.991	0.136
Microvascular invasion (no/yes)	122/80	73/49	49/31	0.04	0.980
Tumor differentiation (grade I/II/III/IV)	4/90/103/5	2/54/64/2	2/36/39/3	1.187	0.978
Ki67 (≦50/>50%)	165/37	101/21	64/16	0.251	0.882
Neutrophil (<4.93/≧4.93 * 10∧9/L)	141/61	85/37	56/24	0.002	0.999
Lymphocyte (<1.06/≧1.06 * 10∧9/L)	23/179	15/107	8/72	0.252	0.882
Platelet (≦192/>192 * 10∧9/L)	93/109	52/70	41/39	1.447	0.485
NLR (<1.23/≧1.23)	25/177	11/111	14/66	3.207	0.201
PLR (<75.1/≧75.1)	44/158	24/98	20/60	0.805	0.669
AFP (<27/≧27 ng/ml)	76/126	49/73	27/53	0.847	0.655
CA125 (<20/≧20 U/ml)	139/63	86/36	53/27	0.405	0.817
CA199 (<5.3/≧5.3 U/ml)	28/174	18/104	10/70	0.206	0.902
HBsAg (no/yes)	32/170	22/100	10/70	1.109	0.574
Child–Pugh (5/6/7/8)	133/58/7/4	80/38/2/2	53/20/5/2	3.784	0.706

*TACE, transarterial chemoembolization; NLR, neutrophil-to-lymphocyte ratio; PLR, platelet-to-lymphocyte ratio; AFP, α-fetoprotein; CA125, carbohydrate antigen 125; CA199, carbohydrate antigen 199*.

### Identification of Prognostic Parameters for SLHCC

**Figure 1:**
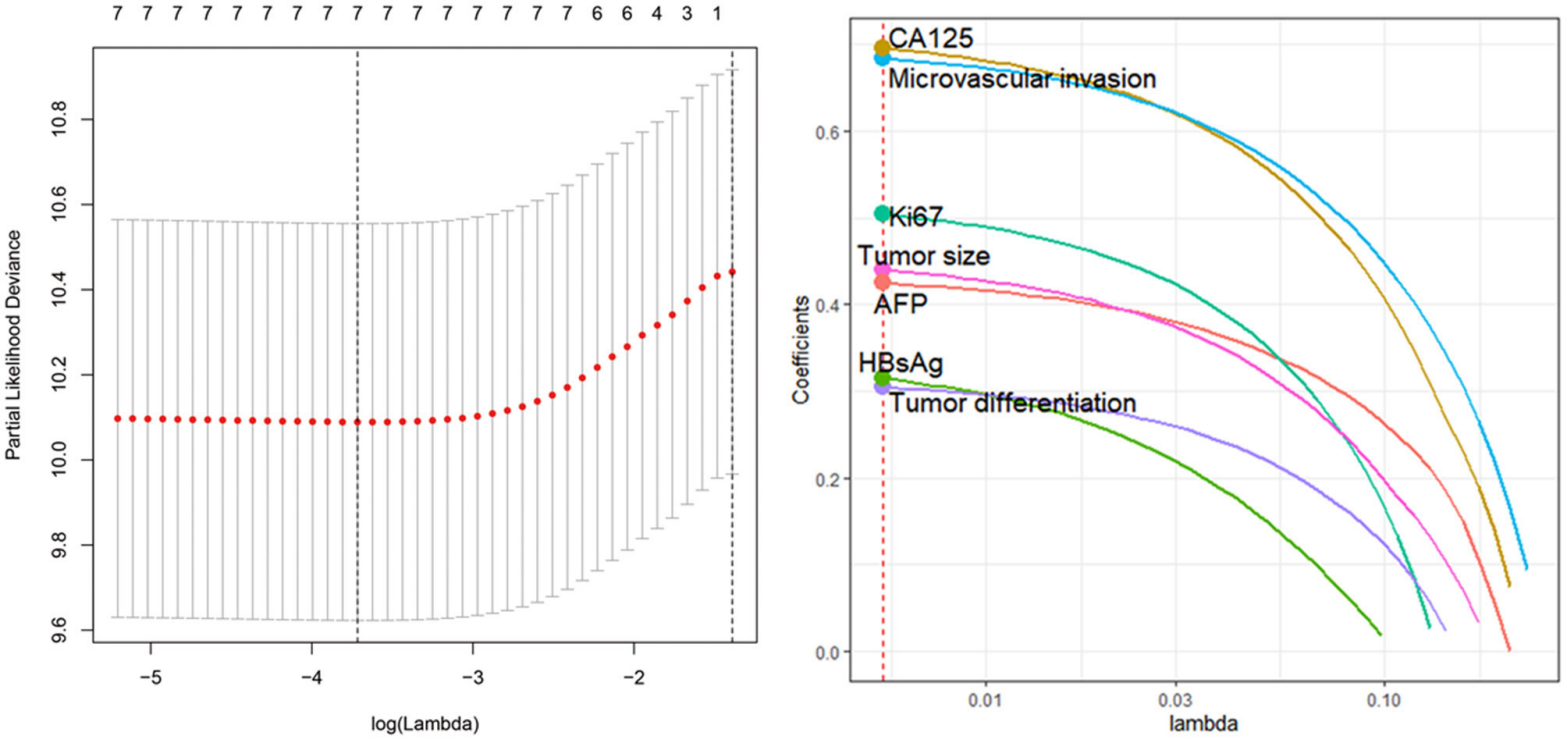
Least absolute shrinkage and selection operator (LASSO) regression analysis identifies seven key prognostic factors for solitary large hepatocellular carcinoma (SLHCC) following curative resection. The seven factors are tumor size, microvascular invasion, tumor differentiation, Ki67 (%), α-fetoprotein (AFP), carbohydrate antigen 125 (CA125), and HBsAg status.

To determine the potential prognostic parameters for SLHCC, we first conducted a univariate Cox regression analysis for OS. The results showed that tumor size (HR = 2.21, 95% CI, 1.30–3.75), microvascular invasion (HR = 2.65, 95% CI, 1.63–4.32), tumor differentiation (HR = 1.82, 95% CI, 1.14–2.90), Ki67 (%) (HR = 1.99, 95% CI, 1.12–3.54), AFP (HR = 2.41, 95% CI, 1.38–4.19), CA125 (HR = 1.13, 95% CI, 1.55–4.23), and HBsAg status (HR = 2.15, 95% CI, 1.02–4.5) were unfavorable prognostic parameters for OS in SLHCC patients (Table 2). In addition, we performed LASSO regression analysis and found that all of these seven prognostic parameters were key prognostic parameters for patients with SLHCC (Figure 1).

**Table 2:** Univariate Cox regression analysis of OS in the training set.

**Parameters**	**HR**	**95% CI**	***P*-value**
Age (≦50/>50 years)	0.85	0.47–1.51	0.575
Gender (female/male)	0.87	0.44–1.71	0.691
Cirrhosis (no/yes)	1.17	0.71–1.95	0.535
Preoperative TACE (no/yes)	0.88	0.32–2.42	0.8
Tumor size (≦7.1/>7.1 cm)	2.21	1.3–3.75	**0.003**
Hepatic capsular invasion (no/invasion but not breaking through/breaking through)	1.25	0.83–1.88	0.287
Microvascular invasion (no/yes)	2.65	1.63–4.32	**8.97E-05**
Tumor differentiation (grade I/II/III/IV)	1.82	1.14–2.9	**0.011**
Ki67 (≦50/>50 %)	1.99	1.12–3.54	**0.019**
Neutrophil (<4.93/≧4.93 * 10∧9/L)	1.56	0.95–2.57	0.08
Lymphocyte (<1.06/≧1.06 * 10∧9/L)	0.76	0.36–1.61	0.48
Platelet (≦192/>192 * 10∧9/L)	0.71	0.44–1.16	0.17
NLR (<1.23/≧1.23)	4.08	1–16.67	0.051
PLR (<75.1/≧75.1)	1.06	0.58–1.95	0.843
AFP (<27/≧27 ng/ml)	2.41	1.38–4.19	**0.002**
CA125 (<20/≧20 U/ml)	2.56	1.55–4.23	**2.36E-04**
CA199 (<5.3/≧5.3 U/ml)	1.13	0.94–1.32	0.053
HBsAg (no/yes)	2.15	1.02–4.5	**0.043**
Child–Pugh (5/6/7/8)	1.2	0.84–1.71	0.314

*TACE, transarterial chemoembolization; NLR, neutrophil-to-lymphocyte ratio; PLR, platelet-to-lymphocyte ratio; AFP, α-fetoprotein; CA125, carbohydrate antigen 125; CA199, carbohydrate antigen 199. Bold values indicates P < 0.05 were considered statistically significant*.

### Establishment of a Novel Prognostic Nomogram for OS for SLHCC

**Figure 2:**
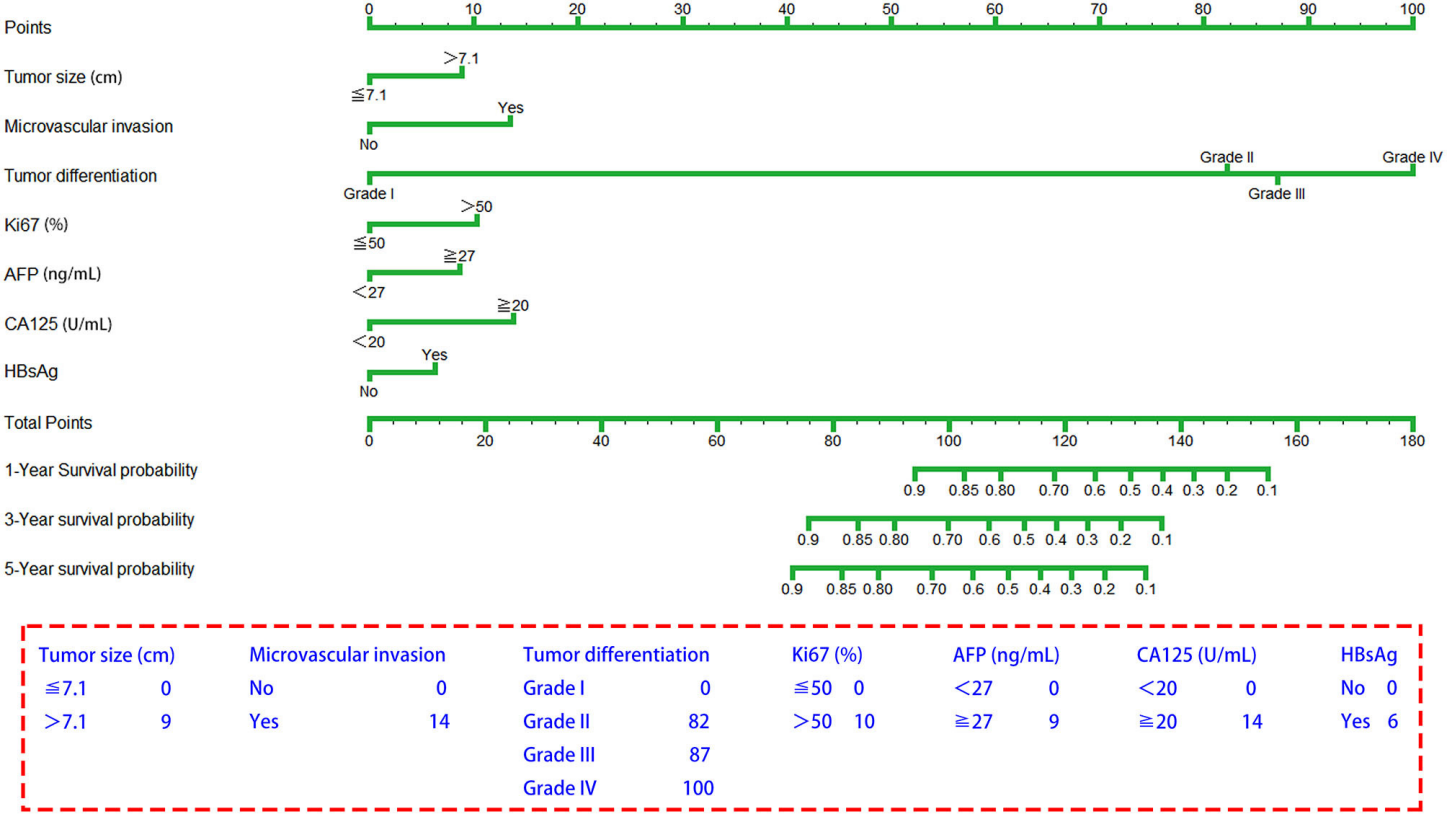
A predictive nomogram is established for SLHCC following curative resection.

**Figure 3:**
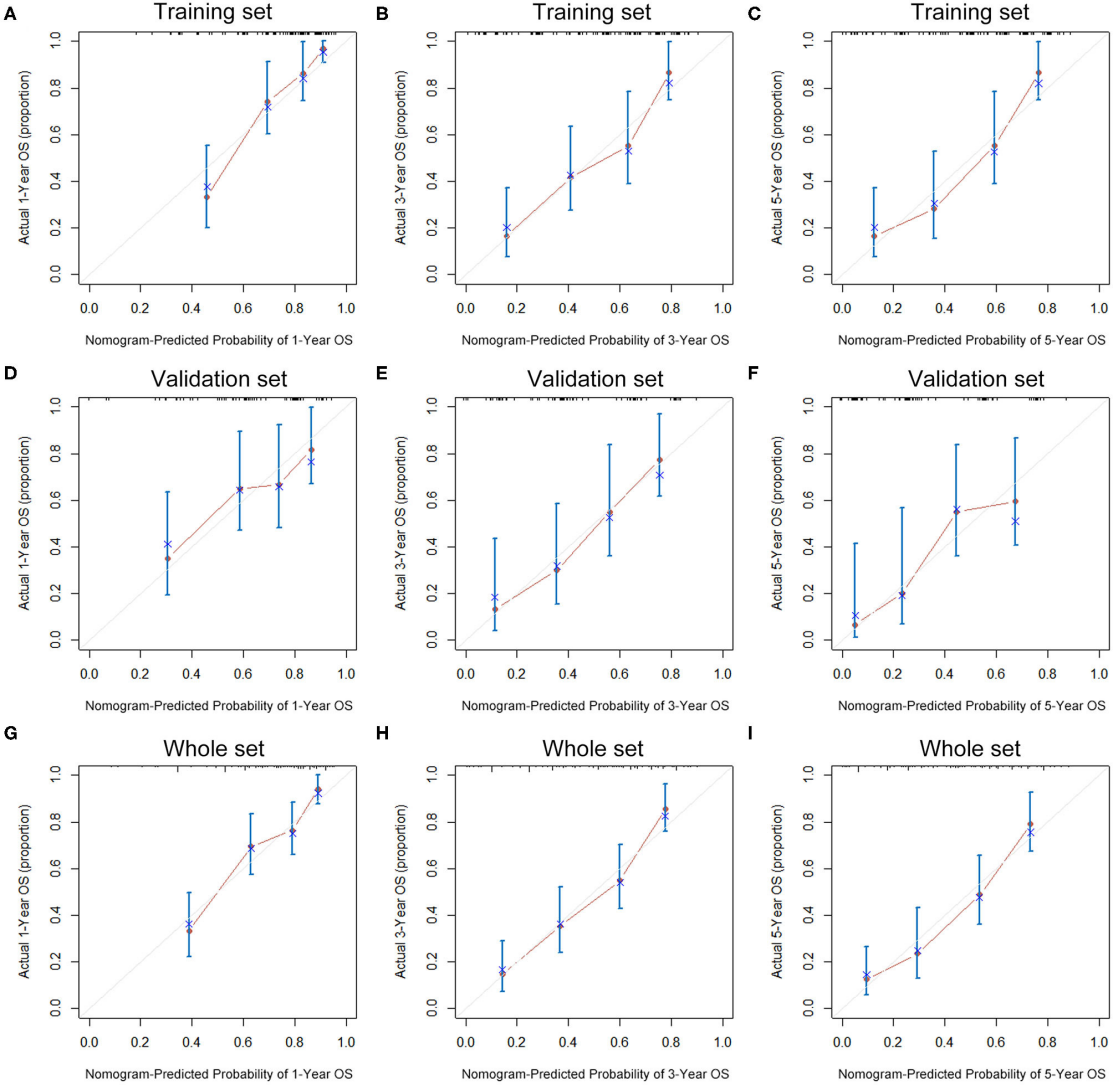
Calibration curves of the nomogram for 1-, 3-, and 5-year OS prediction in the training set **(A–C)**, validation set **(D–F)**, and whole set **(G–I)**.

**Figure 4:**
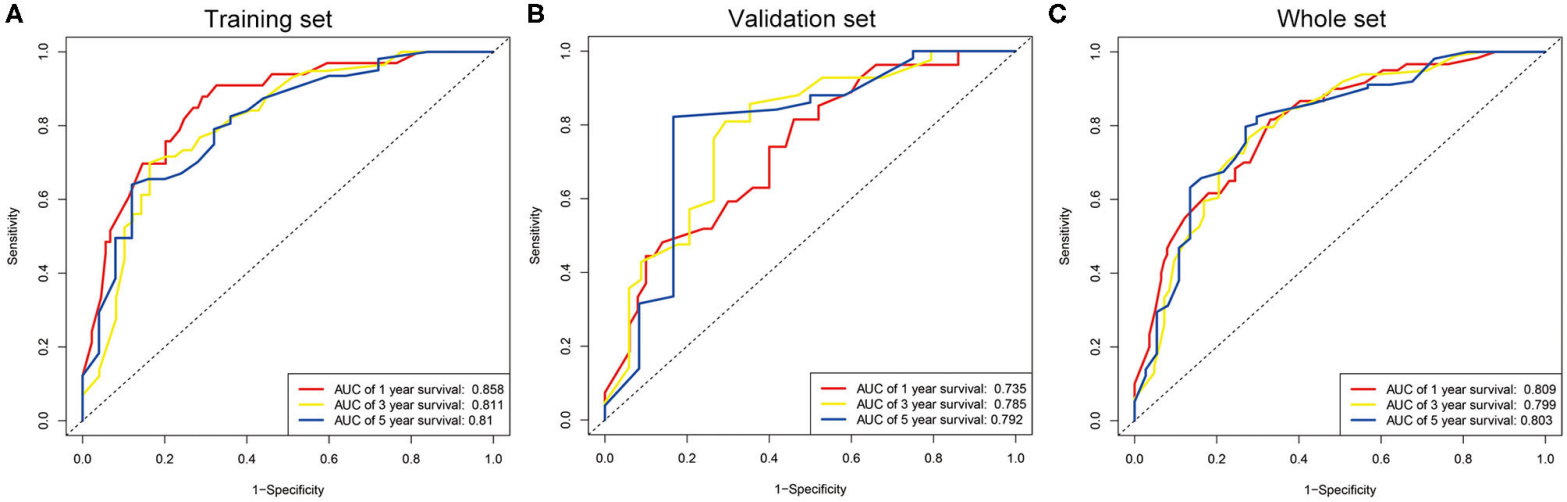
ROC curve analysis of the nomogram for 1-, 3-, and 5-year OS prediction in the training set **(A)**, validation set **(B)**, and whole set **(C)**.

The seven prognostic parameters for OS in the training set were integrated into a newly established nomogram (Figure 2). The C-index of the nomogram for OS prediction in the training set was 0.752 (95% CI, 0.693–0.811). The calibration curves for 1-, 3-, and 5-year OS in the training set presented an optimal agreement between the nomogram-predicted and actual observed survival probabilities (Figures 3A–C). For the validation and whole sets, the nomogram also exhibited a high accuracy of OS prediction, with a C-index of 0.703 (95% CI, 0.625–0.781) in the validation set and a C-index of 0.733 (95% CI, 0.686–0.780) in the whole set. The calibration curves for the prediction of 1-, 3-, and 5-year OS in the validation and whole sets also displayed good agreement (Figures 3D–I). For the training set, the AUC values of the nomogram for predicting 1-, 3-, and 5-year OS were 0.858, 0.811, and 0.810, respectively (Figure 4A). Similarly, the AUC of the nomogram for 1-, 3-, and 5-year OS were 0.735, 0.785, and 0.792 in the validation set, and 0.809, 0.799, and 0.803 in the whole set, respectively (Figures 4B,C). These results indicate an optimal discrimination of prognostic prediction by the nomogram for SLHCC.

We further compared our model with a previously reported prognostic score system for SLHCC, which comprises three factors: tumor size, microvascular invasion, and PLR. The results showed that the C-index of the previously reported score system for OS was 0.626 (95% CI, 0.579–0.673), which was statistically lower than the C-index of our model (C-index = 0.733, 95% CI, 0.686–0.780, *P* < 0.001).

### Prognostic Stratification Based on the Nomogram for SLHCC

**Figure 5:**
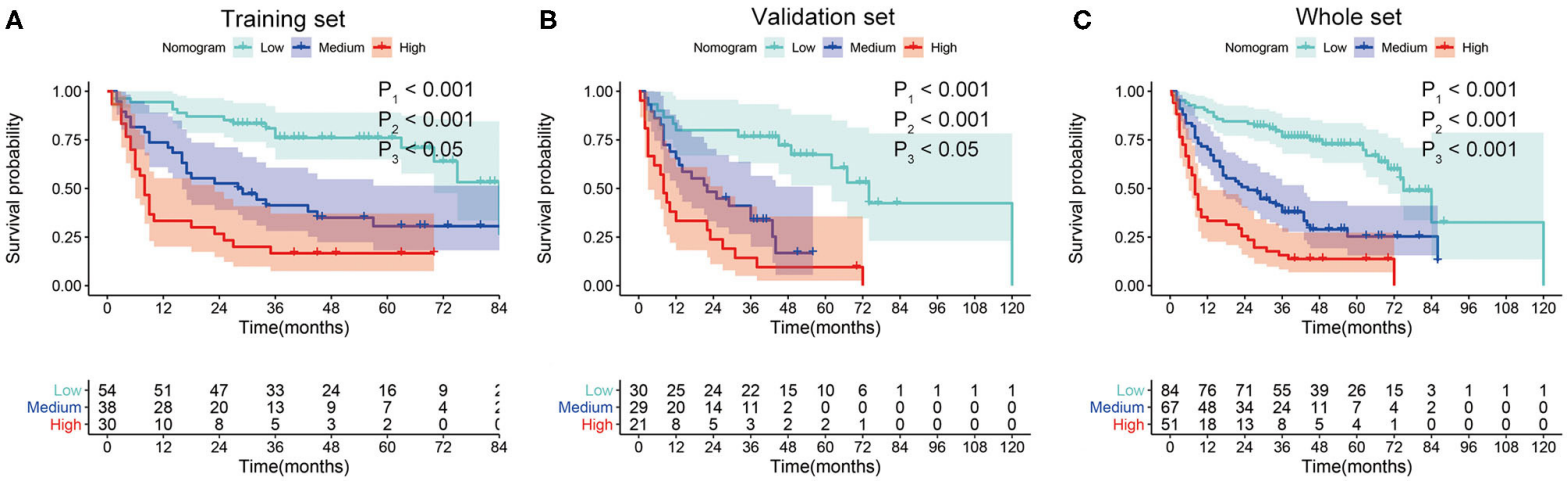
Kaplan–Meier survival curves for overall survival (OS) of patients with SLHCC according to the nomogram-based subgroups in the training set **(A)**, validation set **(B)**, and whole set **(C)**. P1, low-risk vs. medium-risk; P2, low-risk vs. high-risk; P3, medium-risk vs. high-risk.

In order to determine the prognostic stratification classification for SLHCC, we calculated the optimal cutoff points of the nomogram using the X-tile 3.6.1 software in the training set, which were 107 and 125. Thus, the SLHCC patients in the present study were divided into three subgroups: low-risk group (total score ≦ 107), medium-risk group (107 < total score ≤ 125), and high-risk group (total score > 125). The KM survival analysis for OS in the training set showed a significant difference among these three subgroups, in which the low-risk group had the best survival and the high-risk group had the worst survival (Figure 5A). Similar results were also observed in the validation and whole sets (Figures 5B,C). The 1-, 3-, and 5-year OS rates of the low-risk, medium-risk, and high-risk groups in the whole set were 89.3 vs. 70.1 vs. 33.3%, 76.6 vs. 37.8 vs. 14.5%, and 69.8 vs. 25.1 vs. 12.5%, respectively (*P* < 0.001) (Table 3). The median OS of the low-risk, medium-risk, and high-risk groups in the whole set were 75.0 (95% CI, 65.7–84.3) months, 25.0 (95% CI, 12.6–37.4) months, and 8.0 (95% CI, 6.45–9.56) months, respectively (Table 3). The 1-, 3-, and 5-OS rates and median OS of patients in the low-risk group were significantly higher than those in the medium-risk group and high-risk group (*P* < 0.001).

**Table 3:** 1-, 3-, and 5-year OS rates in low-, medium-, and high-risk groups.

	**Whole set**				**Training set**				**Validation set**			
	**Low**	**Medium**	**High**	***P*-value**	**Low**	**Medium**	**High**	***P*-value**	**Low**	**Medium**	**High**	***P*-value**
1-year OS rate	89.30%	70.10%	33.30%	7.77e−16	91.50%	71.80%	32.50%	1.04e−09	80.00%	65.50%	33.30%	2.46e−07
3-year OS rate	76.60%	37.80%	14.50%		76.00%	38.00%	14.00%		76.10%	33.60%	13.30%	
5-year OS rate	69.80%	25.10%	12.50%		75.50%	27.50%	12.40%		66.50%	12.40%	11.50%	
Median OS (months)	75	25	8		84	28	8		74	22	8	

*OS, overall survival*.

**Figure 6:**
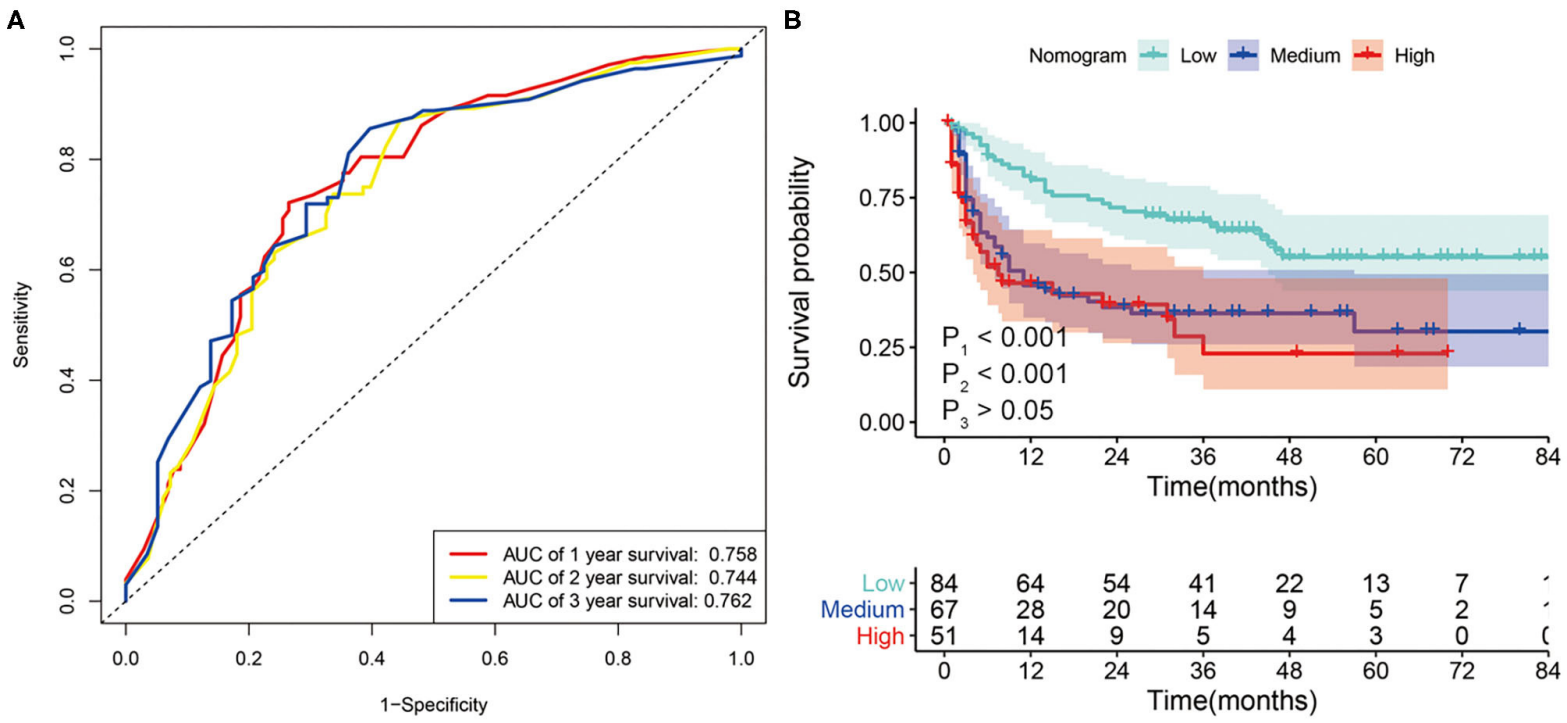
**(A)** ROC curve analysis of the nomogram for 1-, 2- and 3-year recurrence-free survival (RFS) prediction in the whole set. **(B)** Kaplan–Meier survival curves for RFS in patients with SLHCC according to the nomogram-based subgroups in the whole set. P1, low-risk vs. medium-risk; P2, low-risk vs. high-risk; P3, medium-risk vs. high-risk.

We further evaluated the predictive power of the nomogram using tumor recurrence as the endpoint event in the whole set. The AUC of the nomogram for 1-, 2-, and 3-year RFS were 0.758, 0.744, and 0.762, respectively (Figure 6A). Then, we performed KM analysis to evaluate the RFS in the low-risk, medium-risk, and high-risk groups. The 1-, 2-, and 3-year RFS rates of the low-risk, medium-risk, and high-risk groups in the whole set were 81.0 vs. 45.0 vs. 45.5%, 71.0 vs. 37.0 vs. 37.5%, and 66.0 vs. 36.4 vs. 22.9%, respectively. Although no difference was observed between the medium- and high-risk groups, the RFS of patients in the low-risk group was significantly higher than that of the patients in the medium-risk and high-risk groups (*P* < 0.001) (Figure 6B).

### Secondary Treatment Strategy for Tumor Recurrence in SLHCC

**Figure 7:**
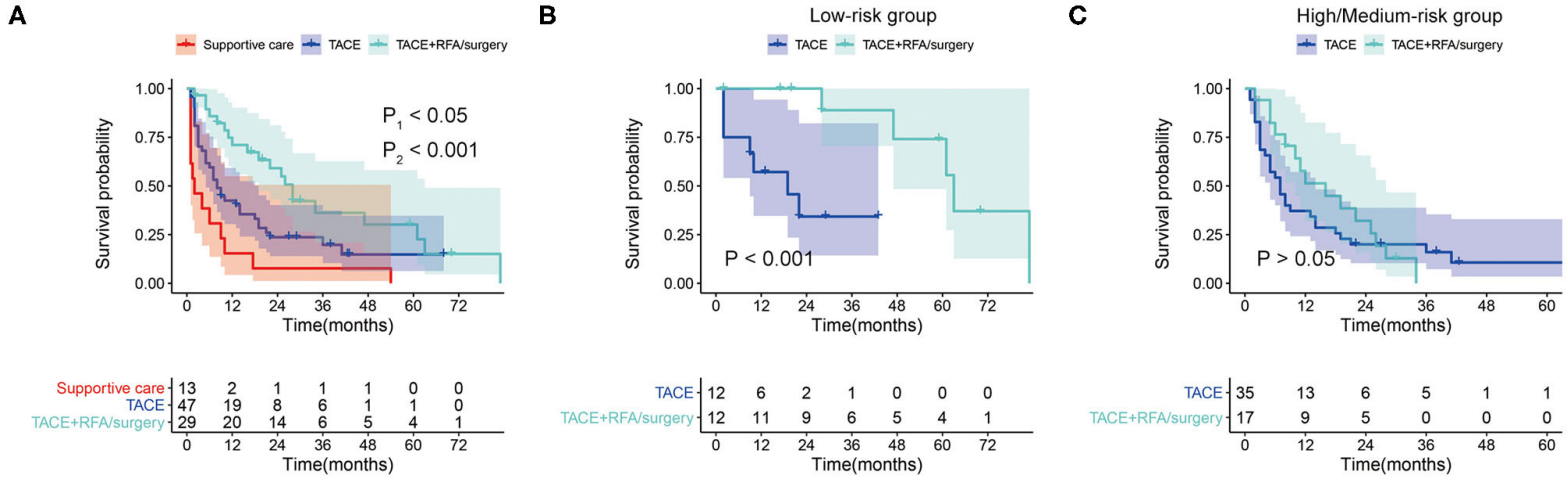
**(A)** Kaplan–Meier survival curves for retreated OS (reOS) in tumor-recurrent patients who received supportive care, transarterial chemoembolizatio (TACE), and TACE plus RFA/surgery (P1, TACE + RFA/surgery vs. TACE; P2, TACE vs. Supportive care). **(B)** Kaplan–Meier survival curves for reOS in tumor-recurrent patients in the low-risk group who received TACE and TACE plus radiofrequency ablation (RFA)/surgery. **(C)** Kaplan–Meier survival curves for reOS in tumor-recurrent patients in the high/medium-risk group who received TACE and TACE plus RFA/surgery.

In the whole set, 101 patients (50%) had tumor recurrence during the follow-up, among which 31 were in the low-risk group, and 70 were in the high/medium-risk group. Of these 101 patients with tumor recurrence, 30 patients received transarterial chemoembolizatio (TACE) plus radiofrequency ablation (RFA)/surgery, 50 patients received TACE only, 3 patients received RFA/surgery only, and 18 patients received supportive care. To explore the effect of the different treatments for recurrent tumors, we investigated retreated OS (reOS), which is the time period from the date of recurrence to the date of death. KM survival analysis demonstrated that patients who received TACE plus RFA/surgery after tumor recurrence had higher reOS compared to TACE alone and supportive care (Figure 7A). The median reOS in the TACE + RFA/surgery, TACE, and supportive care groups were 36.0 (95% CI, 20.3–51.7) months, 17.0 (95% CI, 8.10–25.9) months, and 6.0 (95% CI, 1.84–10.2) months, respectively. Importantly, subgroup analysis showed that TACE plus RFA/surgery significantly prolonged the survival of patients in the low-risk group who suffered from tumor recurrence when compared to TACE alone (Figure 7B). On the contrary, for the high/medium-risk group, no survival advantage was observed in the TACE + RFA/surgery group (Figure 7C).

## Discussion

Although surgical resection is the curative treatment for SLHCC, the outcome could differ between individuals due to distinct tumor biological behavior (11, 16). In the current study, we identified seven unfavorable prognostic factors for OS in patients with SLHCC after curative resection: tumor size, microvascular invasion, tumor differentiation, Ki67 (%), AFP, CA125, and HBsAg status. We next integrated the seven prognostic factors to establish a novel nomogram. The prognostic nomogram showed satisfactory performance for predicting OS and RFS in patients with SLHCC, as evidenced by high C-index and ROC curve analysis in both the training and validation sets. Our study may enable clinicians to accurately predict the prognosis of patients with SLHCC after curative resection and choose a more appropriate postoperative therapy individually.

The prognostic model in our study evaluated and included seven factors, in which tumor size reflects tumor burden, microvascular invasion, Ki67 (%) level, and tumor differentiation, which are pathologic phenotypes, and AFP and CA125 are serum biomarkers. Thus, our model reflects the biological behavior of tumors and the risk of tumor progression. Previously, Shen et al. introduced a prognostic score system consisting of tumor size, microvascular invasion, and PLR for SLHCC (12). To compare the predictive efficacy of their model with ours, we evaluated the scoring system by Shen et al. using the dataset from our institute. The results showed that Shen's model had a C-index of 0.626 (95% CI, 0.579–0.673) for OS, which was statistically lower than the C-index of our nomogram (C-index = 0.733, 95% CI, 0.686–0.780, *P* < 0.001). Of note, tumor differentiation grading contributes the most scores in our nomogram. However, Shen's model did not include tumor differentiation status or other serum biomarkers, which may lead to an inferior predictive power. These results suggest that our prognostic nomogram is more reasonable and exhibits superior predictive value.

In most updated BCLC staging systems, all solitary tumors and two to three nodules ≤ 3 cm are classified as stage A (2). However, recently, some investigators have raised concerns regarding the proper staging of SLHCC. Bruix et al. and Jung et al. found that patients with SLHCC had worse survival rates than patients with solitary HCCs smaller than 5 cm or two to three nodules ≤ 3 cm (17, 18). Therefore, they proposed that it might not be appropriate to classify SLHCC in BCLC stage A. In another study, Tsilimigras et al. subclassified SLHCC as BCLC stage A1, which showed comparable survival with tumors of BCLC stage B (10). In our study, we classified SLHCC into low-risk and medium/high-risk groups based on the prognostic nomogram. We found that the median OS of low-risk SLHCC was nearly five times higher than that of high/medium SLHCC (75.0 vs. 16.0 months, *P* < 0.001). Taken together, it is not suitable to group both the low-risk and high/medium-risk SLHCC in the same stage. Indeed, it should be considered that low-risk SLHCC remains in BCLC stage A, while high/medium-risk SLHCC may be classified as BCLC stage B. Future studies are needed to validate this staging proposal for SLHCC.

Therapeutic options for recurrent HCC include TACE, RFA, repeated surgery, and salvage transplantation (19–22). However, optimal treatment strategies for recurrent HCC after curative resection remain controversial. Only a minor proportion of recurrent HCC is suitable for receiving repeated surgery alone because multiple nodules are commonly seen and patients' liver function is often impaired (19, 23). On the contrary, TACE is easy to perform and is widely used for recurrent HCC. In a previous study, Jin et al. reported that TACE was more effective for recurrent HCC of BCLC 0 or A than RFA/surgery in MVI-positive patients (24). In the current study, only three recurrent cases underwent surgery/RFA alone. In addition, 30 out of 101 patients received TACE plus RFA/surgery, and 50 out of 101 patients received TACE alone. We compared the outcomes of TACE alone and TACE plus RFA/surgery. Interestingly, TACE plus RFA/surgery significantly prolonged the retreated OS when compared to TACE alone in the low-risk group, but not in the medium/high-risk group. These results indicate that recurrent tumors in the medium/high-risk group are more aggressive, and additional RFA/surgery does not have a greater advantage than TACE alone. Thus, we recommend that patients with recurrent SLHCC in the low-risk group receive TACE plus RFA/surgery to eradicate cancer cells at a maximum level. However, for patients in the high-risk group, TACE alone and other supportive care are the main options.

In addition to conventional factors, novel molecular factors may have an important value in prognostic stratification and prediction of treatment response for HCC. Caraglia et al. demonstrated that both the oxidative stress status and pERK activity in peripheral blood mononuclear cells have optimal value in predicting response to sorafenib plus octreotide treatment in advanced HCC patients (25). Additionally, miR-423-5p and fibroblast growth factor receptor 4 have been reported to be effective tools for predicting response to sorafenib or lenvatinib treatment (26, 27). Therefore, it will be interesting to include the critical molecular factors in the prognostic model for SLHCC in future studies.

There are some limitations to our study. First, this was a single-center retrospective study with a relatively small sample size. Second, the study was inherently prone to selection bias due to its retrospective design. Thus, large-scale studies are needed to validate our prognostic model and stratification strategy in the future.

In conclusion, our study develops and validates an effective and reliable nomogram based on seven clinical factors to stratify prognosis in patients with SLHCC after curative resection, which provides an important rationale for prospective randomized controlled trial design. The prognostic value of novel molecular factors deserves further investigation.

## Data Availability Statement

The original contributions presented in the study are included in the article/supplementary material, further inquiries can be directed to the corresponding author/s.

## Ethics Statement

The protocol of this study was approved by the Clinical Research Ethic Committee of Guangdong Provincial people's Hospital. Written informed consent to participate in this study was provided by the patients.

## Author Contributions

H-KZ and C-ZZ: study conception, design, quality control of this study, drafting of the manuscript, and statistical analysis. Z-XZ, Z-YM, S-ZH, Y-FG, and Z-DZ: data acquisition and data interpretation. C-ZZ, W-XY, and B-HH: resource and study supervision. All authors have read and approved the final manuscript.

## Conflict of Interest

The authors declare that the research was conducted in the absence of any commercial or financial relationships that could be construed as a potential conflict of interest.

## References

[1] McGlynn KA Petrick JL El-Serag HB Epidemiology of hepatocellular carcinoma. Hepatology. (2020). 10.1002/hep.31288 PMC7577946 32319693

[2] European Association for the Study of the Liver Electronic address eee, European Association for the Study of the L. EASL clinical practice guidelines: management of hepatocellular carcinoma. J Hepatol. (2018) 69:182–236. 10.1016/j.jhep.2018.03.019 29628281

[3] Forner A Reig ME de Lope CR Bruix J. Current strategy for staging and treatment: the BCLC update and future prospects. Semin Liver Dis. (2010) 30:61–74. 10.1055/s-0030-1247133 20175034

[4] Forner A Reig M Bruix J. Hepatocellular carcinoma. Lancet. (2018) 391:1301–14. 10.1016/S0140-6736(18)30010-2 29307467

[5] Lunse S Heidecke CD Partecke LI. Current topics and perspectives in surgical management of hepatocellular carcinoma. In: Tirnitz-Parker JEE editor. Hepatocellular Carcinoma. Brisbane, QLD: Codon Publications (2019). p. 111–25. 31664800

[6] Yang LY Fang F Ou DP Wu W Zeng ZJ Wu F Solitary large hepatocellular carcinoma: a specific subtype of hepatocellular carcinoma with good outcome after hepatic resection. Ann Surg. (2009) 249:118–23. 10.1097/SLA.0b013e3181904988 19106686

[7] Goh BK Teo JY Chan CY Lee SY Jeyaraj P Cheow PC Importance of tumor size as a prognostic factor after partial liver resection for solitary hepatocellular carcinoma: implications on the current AJCC staging system. J Surg Oncol. (2016) 113:89–93. 10.1002/jso.24099 26611492

[8] Esnaola NF Lauwers GY Mirza NQ Nagorney DM Doherty D Ikai I. Predictors of microvascular invasion in patients with hepatocellular carcinoma who are candidates for orthotopic liver transplantation. J Gastrointest Surg. (2002) 6:224–32; discussion: 232. 10.1016/S1091-255X(01)00015-4 11992808

[9] Pawlik TM Delman KA Vauthey JN Nagorney DM Ng IO Ikai I. Tumor size predicts vascular invasion and histologic grade: implications for selection of surgical treatment for hepatocellular carcinoma. Liver Transpl. (2005) 11:1086–92. 10.1002/lt.20472 16123959

[10] Tsilimigras DI Bagante F Sahara K Moris D Hyer JM Wu L. Prognosis after resection of barcelona clinic liver cancer (BCLC) stage 0, A, and B hepatocellular carcinoma: a comprehensive assessment of the current BCLC classification. Ann Surg Oncol. (2019) 26:3693–700. 10.1245/s10434-019-07580-9 31267302

[11] Yang LY. The progresses of liver resection for solitary large hepatocellular carcinoma. Zhonghua Wai Ke Za Zhi. (2020) 58:13–16. 10.3760/cma.j.issn.0529-5815.2020.01.004 31902163

[12] Shen JY Li C Wen TF Yan LN Li B Wang WT. A simple prognostic score system predicts the prognosis of solitary large hepatocellular carcinoma following hepatectomy. Medicine. (2016) 95:e4296. 10.1097/MD.0000000000004296 27495033 PMC4979787

[13] Liu PH Su CW Hsu CY Hsia CY Lee YH Huang YH. Solitary large hepatocellular carcinoma: staging and treatment strategy. PLoS ONE. (2016) 11:e0155588. 10.1371/journal.pone.0155588 27176037 PMC4866714

[14] Camp RL Dolled-Filhart M Rimm DL. X-tile: a new bio-informatics tool for biomarker assessment and outcome-based cut-point optimization. Clin Cancer Res. (2004) 10:7252–9. 10.1158/1078-0432.CCR-04-0713 15534099

[15] Iasonos A Schrag D Raj GV Panageas KS. How to build and interpret a nomogram for cancer prognosis. J Clin Oncol. (2008) 26:1364–70. 10.1200/JCO.2007.12.9791 18323559

[16] Wei CY Chen PC Chau GY Lee RC Chen PH Huo TI. Comparison of prognosis between surgical resection and transarterial chemoembolization for patients with solitary huge hepatocellular carcinoma. Ann Transl Med. (2020) 8:238. 10.21037/atm.2019.12.157 32309385 PMC7154415

[17] Bruix J Llovet JM. Prognostic prediction and treatment strategy in hepatocellular carcinoma. Hepatology. (2002) 35:519–24. 10.1053/jhep.2002.32089 11870363

[18] Jung YK Jung CH Seo YS Kim JH Kim TH Yoo YJ. BCLC stage B is a better designation for single large hepatocellular carcinoma than BCLC stage A. J Gastroenterol Hepatol. (2016) 31:467–74. 10.1111/jgh.13152 26332049

[19] Zhang X Li C Wen T Yan L Li B Yang J. Appropriate treatment strategies for intrahepatic recurrence after curative resection of hepatocellular carcinoma initially within the Milan criteria: according to the recurrence pattern. Eur J Gastroenterol Hepatol. (2015) 27:933–40. 10.1097/MEG.0000000000000383 25933127

[20] Tung-Ping Poon R Fan ST Wong J. Risk factors, prevention, and management of postoperative recurrence after resection of hepatocellular carcinoma. Ann Surg. (2000) 232:10–24. 10.1097/00000658-200007000-00003 10862190 PMC1421103

[21] Zhang X Li C Wen T Peng W Yan L Yang J. Outcomes of salvage liver transplantation and re-resection/radiofrequency ablation for intrahepatic recurrent hepatocellular carcinoma: a new surgical strategy based on recurrence pattern. Dig Dis Sci. (2018) 63:502–14. 10.1007/s10620-017-4861-y 29238896

[22] Song Q Ren W Fan L Zhao M Mao L Jiang S Long-term outcomes of transarterial chemoembolization combined with radiofrequency ablation vs. transarterial chemoembolization alone for recurrent hepatocellular carcinoma after surgical resection. Dig Dis Sci. (2020) 65:1266–75. 10.1007/s10620-019-05733-0 31312995

[23] Minagawa M Makuuchi M Takayama T Kokudo N. Selection criteria for repeat hepatectomy in patients with recurrent hepatocellular carcinoma. Ann Surg. (2003) 238:703–10. 10.1097/01.sla.0000094549.11754.e6 14578733 PMC1356149

[24] Jin YJ Lee JW Lee OH Chung HJ Kim YS Lee JI Transarterial chemoembolization vs. surgery/radiofrequency ablation for recurrent hepatocellular carcinoma with or without microvascular invasion. J Gastroenterol Hepatol. (2014) 29:1056–64. 10.1111/jgh.12507 24372785

[25] Caraglia M Giuberti G Marra M Addeo R Montella L Murolo M. Oxidative stress and ERK1/2 phosphorylation as predictors of outcome in hepatocellular carcinoma patients treated with sorafenib plus octreotide LAR. Cell Death Dis. (2011) 2:e150. 10.1038/cddis.2011.34 21525937 PMC3122065

[26] Stiuso P Potenza N Lombardi A Ferrandino I Monaco A Zappavigna S. MicroRNA-423-5p promotes autophagy in cancer cells and is increased in serum from hepatocarcinoma patients treated with sorafenib. Mol Ther Nucl Acids. (2015) 4:e233. 10.1038/mtna.2015.8 25782064

[27] Yamauchi M Ono A Ishikawa A Kodama K Uchikawa S Hatooka H. Tumor fibroblast growth factor receptor 4 level predicts the efficacy of lenvatinib in patients with advanced hepatocellular carcinoma. Clin Transl Gastroenterol. (2020) 11:e00179. 10.14309/ctg.0000000000000179 32677805 PMC7263646

